# Solution of Levinthal’s Paradox and a Physical Theory of Protein Folding Times

**DOI:** 10.3390/biom10020250

**Published:** 2020-02-06

**Authors:** Dmitry N. Ivankov, Alexei V. Finkelstein

**Affiliations:** 1 Center of Life Sciences, Skolkovo Institute of Science and Technology, 121205 Moscow, Russia; 2 Institute of Protein Research, Russian Academy of Sciences, 142290 Pushchino, Moscow Region, Russia; 3 Biology Department, Lomonosov Moscow State University, 119192 Moscow, Russia; 4 Biotechnology Department, Lomonosov Moscow State University, 142290 Pushchino, Moscow Region, Russia

**Keywords:** protein folding, Levinthal’s paradox, “all-or-none” transition, free energy barrier, folding funnel, detailed balance principle

## Abstract

“How do proteins fold?” Researchers have been studying different aspects of this question for more than 50 years. The most conceptual aspect of the problem is how protein can find the global free energy minimum in a biologically reasonable time, without exhaustive enumeration of all possible conformations, the so-called “Levinthal’s paradox.” Less conceptual but still critical are aspects about factors defining folding times of particular proteins and about perspectives of machine learning for their prediction. We will discuss in this review the key ideas and discoveries leading to the current understanding of folding kinetics, including the solution of Levinthal’s paradox, as well as the current state of the art in the prediction of protein folding times.

## 1. Introduction

The problem of protein folding is one of the most important problems of modern theoretical biophysics. It appeared more than 50 years ago when Anfinsen et al. demonstrated that proteins can spontaneously fold into their unique 3D native structure in vitro [1,2]. (We will mostly speak about single-domain water-soluble globular proteins, mainly of ~20–300 amino acid residues, or separate water-soluble globular domains of multi-domain proteins, because most of the research on spontaneous protein folding has been done on that kind of protein molecules. We will not consider membrane and fibrous proteins, which, as a rule, experience an assisted folding rather than spontaneous self-organization.) Since Anfinsen’s experiments, the problem of protein self-organization attracted much attention of physicists and became the major problem of protein physics (sometimes called, after E.I. Shakhnovich, the “Fermat’s Last Theorem of protein physics”).

The protein folding problem is an umbrella term for a dozen related problems coupled with protein folding. Here, we will review the history of investigation and solution of the conceptually major and the most puzzling question: How can proteins confidently find their unique 3D native structure among zillion alternatives in a biologically reasonable time? We will leave aside the other problems, such as prediction of protein 3D native structure from protein sequence, which has an applied character and is still unsolved in a general case [3], despite the substantial recent progress based on usage of the multiple deep neural networks, see [4,5].

In this review, we shall consider mostly the best-studied (both experimentally and theoretically) case of the in vitro folding of relatively small (single-domain) water-soluble globular proteins.

However, it is not out of place to first consider the similarities and differences between in vivo and in vitro protein folding. Fortunately, this can be done rather readily, because a recent review published in *Biomolecules* [6] outlines progress in the field of in vivo protein folding. (See also References [7,8].)

We can begin by comparing protein structure formation times in vivo and in vitro. For in vivo folding, the average time elapsed during protein synthesis has been measured at about 0.1 s per amino acid residue, both in bacterial and eukaryotic cells [9,10], while the spontaneous folding time of single-domain globular proteins in vitro ranges from a fraction of a microsecond per residue for small proteins to many seconds per residue for large single-domain globular proteins. (See Supplement Table to Reference [11].) This means that protein folding in vitro can be both faster and slower than in vivo protein biosynthesis.

Second, a number of experiments on large proteins have shown that their N-terminal domains are able to fold before the biosynthesis of the whole chain has been completed [12]. In addition, it has been shown that the multi-domain protein luciferase folds during biosynthesis or immediately after it [13,14]. It has also been shown that a chain of staphylococcal nuclease that is truncated (from the C-end) is compact but disordered [15], but a globin chain can efficiently bind the heme when only a little more than half of the chain has been synthesized by the ribosome [16]. It is not clear, however, if this truncated globin chain folds before interaction with the heme or as a result of this interaction. (If folding were to result from the interaction, that behavior would resemble that of intrinsically disordered proteins interacting with their ligands [17,18].)

Co-translational folding can occur early, within the polypeptide exit tunnel, and/or at the surface of the ribosome; the interaction with the ribosome can alter the folding trajectory (as compared to that of in vitro folding) in many different ways [6]. For instance, ribosomes can facilitate protein compaction and induce the formation of intermediates that are not observed in the solution. Some further examples of this are summarized in the review [6].

However, it seems that there is no fundamental difference between the in vivo (co-translational) folding and in vitro refolding of denatured proteins, at least not for small, single-domain globular proteins: In both cases, three-dimensional native protein structures emerge only after the entire sequence is available. Experiments on co-translational structure acquisition of small nascent protein chains (monitored by NMR and FRET methods) have shown that “polypeptides remain unstructured during [co-translational] elongation [at a ribosome] but fold into a compact, native-like structure when the entire sequence is available” [19] and that “co-translational folding … proceeds through a compact, non-native conformation … [and] rearranges into a native-like structure immediately after the full domain sequence has emerged from the ribosome” [20]. The same effect has been observed for co-translational folding within a mammalian cell [21]. Moreover, phi-value analysis [12], which allows one to localize the protein folding nucleus (a key “barrier” structure, i.e., “bottleneck” in the folding pathway), suggests that the nucleus of the Ig domain is conserved when the folding occurs on and off the ribosome [22]. It is noteworthy, however, that the observed metastable intermediates of folding of some small proteins, although they are not native-like, can be rather different during in vivo than during in vitro protein folding [6]. It is also noteworthy that co-translational folding can sometimes alleviate the need for chaperone assistance [13].

In this review, we will not consider folding of multiple-domain proteins, whose co-translational folding seems to be facilitated by the sequential appearance of the nascent protein chain from the ribosome. Nor will we consider membrane, fibrous, or oligomeric proteins. (See References [23,24,25] for recent reviews.) That is because not all of these proteins are capable of spontaneous folding (renaturation). Rather, we shall concentrate on water-soluble globular proteins that can spontaneously form their unique 3D native structures in vitro [1,2].

Since these proteins fold into their native structures at proper ambient conditions independently of initial states of their chains, there should be something special about them from the physical point of view. Anfinsen proposed a “thermodynamic hypothesis”, assuming that the native structure is the global free energy minimum at physiological conditions [2] because all chains of a given protein fold into the same native structure in diverse processes: At biosynthesis, after renaturation, or even after chemical synthesis [26]. (We consider only conformations of separate monomeric protein chains, and do not consider aggregated structures considered in [7]).

Then, a protein has to find the native structure within biologically relevant time (minutes) and be sure that the found structure is the global free energy minimum. In order to prove that the found structure is the most stable, Levinthal considered a scenario when the protein has to probe all the conformations, check their free energies, and choose the most stable conformation [27,28]. However, the overall number of conformations is astronomically huge: At least 2^100^ for a typical globular domain of 100 amino acid residues since at least two different conformations exist for an amino acid residue in a protein chain. If the protein chain changes its conformation every picosecond (time of thermal vibration, the fastest physical process at room temperature), then the sampling of all 2^100^ conformations would take ~10^10^ years. (An experiment shows that an amino acid residue of a protein chain can accept ten different conformations, on average [29]. Originally, Levinthal assumed that an amino acid residue in protein has even 100 different conformations, having ten possible values of ϕ and ten possible values of ψ angle [28].) Additionally, at first glance, there is no way to substantially reduce the number of conformations to sample because the folding landscape seems to be very rugged: Even a 1 Å deviation of residue side chain can strongly decrease the stability of a densely packed protein conformation.

Therefore, we have a paradox. If the native structure is a global free energy minimum, then a protein has to check all the conformations to be sure that the native conformation is indeed the global minimum. On the other hand, proteins do not have billion years to sample all the conformations since proteins have to fold within minutes, maximum in hours. Therefore, concluded Levinthal, (i) contrary to Anfinsen’s “thermodynamic hypothesis”, the native structure does not need to be the global free energy minimum, (ii) protein folding follows some fast special pathway invented and maintained by the evolution, and (iii) the native structure is just the end of that special pathway. In other words, Levinthal postulated kinetic control of protein folding because he could not reconcile kinetics (fast folding pathway) with thermodynamics (folding into the global minimum of free energy).

However, from the experimental data, we **expect** the protein native structure to be the global free energy minimum because, as mentioned above, this structure is obtained as a result of different folding processes. We also see that proteins fold fast into their native structures. It seems that there is no conflict between kinetics and thermodynamics; they agree with each other in real life. Conducting the direct experiment to resolve the conflict (if any) is impossible: Assume we want to check the presence of a structure which is more stable than the native one; should we wait for 10^10^ years?

As for the computer experiments, it was shown in 1994 that model protein chains do fold fast to the structure corresponding to the global free energy minimum, provided it is at least by a few kcal/mol deeper than the other free energy minima, i.e., in this case, the protein folding obeys thermodynamic control [30,31]. This confirms the absence of conflict between “kinetics control” and “thermodynamics control” for sufficiently stable protein structures and hints that fast folding pathway to the global free energy minimum automatically exists. However, why must such a pathway exist? What are the characteristics of such a pathway? Finally, how do proteins fold? These and other questions address protein folding kinetics. Therefore, a protein folding model has to present a kinetic explanation of protein folding. Probably, that was the reason why most of the protein folding models and/or hypotheses utilized the “kinetic control” assumption:

First, Phillips in 1966 proposed that protein starts its folding using a structure formed by its N-terminal residues [32], which are the first to emerge from a protein synthesizing ribosome, and when the remaining protein chain comes from the ribosome, it wraps around this first-formed “seed” structure. However, many proteins fold after a circular permutation that moves the N-terminal piece of its chain to the C-end of the chain [33]. Thus, the N-end of the chain plays no special role.

Second, Wetlaufer hypothesized that the adjacent residues should form the native-like “seed” structure first [34]. However, numerous later experiments showed that it is not necessary for successful folding.

Third, Ptitsyn proposed a model of hierarchical folding, where native-like elements of secondary structure fold first, and only after that, the tertiary structure appears [35].

Finally, the “folding funnel” concept became extremely popular to illustrate and give a kind of explanation of protein folding [36,37,38,39] (we will discuss folding funnels below).

Note that the question “What governs protein folding, kinetics or thermodynamics?” is not purely scholastic; it has a significant practical impact. For example, when designing a protein de novo, should we try to design high stability of the native structure or a fast folding pathway to it? When predicting 3D native structure from the amino acid sequence, should we look for the most stable or for the most rapidly folding structure?

Let us refresh some basic experimental facts about **thermodynamics** and **kinetics** of folding and denaturation of single-domain proteins. These facts will help us understand what chains and what folding conditions we have to consider. The facts are as follows:

1. Although a denatured protein chain can be in a number of thermodynamic states (such as molten globule and random coil), transitions between these states are much less pronounced than transition between any of them and the native protein globule; a strong denaturant can bring proteins to the state of the completely unfolded random coil [40].
2. Unfolding and folding of separate protein molecules are reversible processes [2,12,41]; we will not consider protein aggregation, which can only prevent this reversibility.
3. Both these processes, unfolding and folding, are observed even at the mid-transition (i.e., equilibrium) point, where the native and unfolded states have equal stabilities [12,41].
4. The transition between the native and denatured structure is an “all-or-none” transition [29], meaning that only native and denatured states are observed to any detectable quantity, while all alternative structures, “half-folded” or “misfolded”, are virtually absent. (Note that: (i) The "all-or-none" transition ensures the robustness of protein functioning: Like a light bulb, the protein either works or not; (ii) the physical theory shows that the "all-or-none" transition requires the protein sequence to provide a sufficiently large "energy gap" between the most stable structure and the bulk of misfolded ones [31,42,43,44,45].)
5. Even at physiological conditions, the native (i.e., the most stable) state of a protein is just slightly more stable than its unfolded or any other denatured state of the separate protein molecule in solution. The difference in their stabilities constitutes only a few kilocalories per mole [29], which is much less than the energy of the native relatively to the denatured state. Additionally, at the mid-transition (i.e., equilibrium) point, the native and unfolded states have, obviously, equal free energies and equal stabilities.

The “all-or-none” transition means that a sufficiently high free-energy barrier separates the native and denatured states. It is the height of this barrier that defines the kinetics of protein folding and unfolding. Thus, just the barrier height is to be estimated to elucidate the Levinthal’s paradox.

## 2. Folding Funnels per se do not Solve Levinthal’s Paradox

Now, it is reasonable to take a closer look at the Levinthal’s paradox from the physical point of view, that is, to build a physical model corresponding to the Levinthal’s model. Maybe, the closer look will show that there is nothing physically paradoxical in this paradox at all! Indeed, the Levinthal’s model looks more like a computer program: Protein goes one-by-one through the ordered list of conformations, somehow remembers the most stable conformation, and then comes back to the best choice. The first strange thing here is, how can a protein order the list of conformations? The second is, how can a protein remember the most stable conformation and come back to it at the end? Too smart for a protein, right?

**Figure 1:**
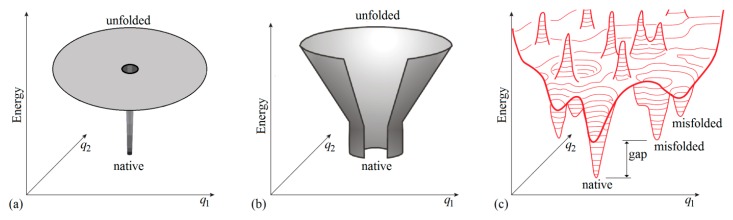
Illustrations of the protein energy landscape for different models. For illustrative purposes, the coordinates *q_1_* and *q_2_* schematically present the protein conformational space, which has, in reality, hundreds of dimensions. (**a**) The energy landscape for the “golf course” model. (**b**) A general “energy funnel” illustration. The funnel centers on the protein native structure having the lowest energy. (We should remind that the term "entropy" in the context of protein folding models conventionally refers only to the configurational entropy, while the term "energy" refers to the free energy of interactions, so that solvent-mediated forces (for example, hydrophobic and electrostatic ones), with all their solvent entropy [40] come within "energy"; therefore, we avoid applying the word “enthalpy” to protein folding models.) (**c**) A more detailed general energy landscape. The term “gap” denotes an energy gap between the global and other energy minima, necessary to provide the “all-or-none” transition between native and other structures of the chain. The gap must be large enough, of many *k*_B_*T*_melt_, where *T*_melt_ is protein melting temperature (thus, usually, *k*_B_*T*_melt_ ≈ 0.7 kcal/mol). Adapted from [46] with some modifications.

The physical model most closely matching the Levinthal’s is the “golf course” model (Figure 1a) [38]. Here, a protein like a ball rolling over a flat landscape, samples conformations randomly until it hits the golf hole that is the native structure. To better imagine the protein conformational sampling problem, note that if we would build the golf field following Figure 1a, its size (covering the area of 2^100^ golf holes) would be much bigger than the solar system. The average time of hitting the native structure in the golf course model is the same as in Levinthal’s model. A small technical difference is that in the original Levinthal’s model, a protein **must** check all the conformations, and thus, the time for every folding run is exactly 2^100^ ps. In the golf course model, the most probable (and the average) time is 2^100^ ps, but the time of a run may be somewhat shorter or larger due to random sampling.

Unlike the ball in golf, the protein has an opportunity to escape from the native structure due to the thermal movement characterized by thermal energy *k*_B_*T* (*k*_B_ is the Boltzmann constant, *T* is the absolute temperature of the medium). However, the free energy barrier between the native (hole) and unfolded (flat surface) states in the “golf course” model is inevitably high [47]: The native protein structure has to lose all its negative stabilizing energy (that is, thermal movements push it out of the native “hole”) before it gains the entropy of the unfolded state. Vice versa, during folding, the protein has to lose first (**before** falling into the hole) all its entropy, and only after that, it gains the native structure stability. For our future consideration note also that the energy of the native state, as well as the unfolded state entropy, and therefore the free-energy barrier in the “golf course” model is proportional to the size of the protein, i.e., to the number of amino acid residues *L*. However, a separation of the unfolded and native-like phases during the folding creates much lower barriers (see below).

Overall, the problem of huge sampling comes from the “combinatorial explosion”; finding the lowest free-energy conformation of a protein by sampling is an “NP-hard” problem (a problem which, in general, cannot be solved in a time polynomial with respect to *L*), as it has been proved mathematically [48,49]. This, loosely speaking, means that the problem requires an exponentially big time for its solution, by a man or by a protein chain. Usually, one takes “NP-hard” as “endlessly long”, but we will show (see below) that although the protein folding problem is NP-hard indeed, it can be solved in a reasonable time for such chains with *L* ≲ 500 that allow a separation of the unfolded and native-like phases during the folding, which creates a **special** kind of funnels, i.e., **funnels with phase separation**.

It is interesting that the stepwise mechanism proposed by Ptitsyn [35], taken per se, also cannot **simultaneously** explain (1) nonastronomical folding time, and (2) co-existence of folding and unfolding processes close to the mid-transition point [12,41]. In Ptitsyn’s model, the most stable structure built at each step serves as a seed for the even more stable next-step structure. This helps to avoid a huge sampling of the conformational space. However, the thermodynamic stability of the intermediate structures constantly increases on the course of folding, meaning that the native structure is much more stable than the unfolded one. Such a scenario can work only far from the point of thermodynamic equilibrium and does not explain the observed [12,41] folding at the mid-transition point.

Thus, although the considered theories captured some of the important features of protein folding, they do not solve the Levinthal’s paradox. The solution of the Levinthal paradox for the simplest case [50,51,52] relies on the nucleation–condensation mechanism, where the key role is played by the separation of the unfolded and native phase. We will consider the solution of Levinthal’s paradox in the next part of the paper.

## 3. Nucleation-Based Physical Theory Solves the Levinthal’s Paradox 

The major prerequisite for the building of the physical theory is to consider folding at the mid-transition point where folding looks the simplest. Protein folding has the following crucial peculiarities here:

1. The native protein structure folds and unfolds reversibly, and, by definition, the unfolded state of the chain is as stable as the native protein state at the mid-transition point, where the energy of the native state compensates the entropy of the unfolded state.
2. The native structure folds and unfolds through an “all-or-none” transition, which means that the free energy barrier separates the native protein from the ensemble of unfolded structures [29]. Without the barrier, a protein chain of ~100 residues would fold within a microsecond because the experimentally measured elementary time of inclusion of a residue into growing secondary structure is τ ~ 1–10 ns [53] (this elementary time τ includes, of course, all femto- and picosecond rearrangements of liquid water around polypeptides). Proteins of ~100 residues fold within millions or billions of microseconds because they spend almost all the folding time *t* attempting to overcome the free energy barrier. According to the transition state theory [54], the time to overcome a free-energy barrier of the height ∆*G*^#^ at temperature *T* is
  *t* ~ τ × exp(+∆*G*^#^*/RT*). (1)
3. The “all-or-none” transition implies the existence of a substantial energy gap between the native protein and alternative misfolded structures [31,42,43,44,45]. Thus, when the unfolded and the natively folded states are equally stable, we can ignore folding into misfolded alternative structures because their free energy is very high and thus their population at the mid-transition point is invisibly low, so that they cannot compete with correct folding.
4. According to the physical “detailed balance principle” [55] (following from the impossibility of a “perpetual motion machine of the second kind”, which, in turn, is a direct consequence of the Second Law of thermodynamics: such a machine would be possible, if a pathway of direct reaction were different, under the same equilibrium ambient conditions, from the pathway of recurrent reaction, because this difference would lead to a perpetual directed flow under equilibrium ambient conditions, and this flow would be able to power the perpetual motion machine of the second kind), the distribution, probabilities, and rates of the folding pathways are the same as those of the unfolding pathways (under the same equilibrium ambient conditions, of course), but going in the reverse direction. (If ambient conditions under which the direct reaction (say, folding) is studied differ from the ambient conditions under which the recurrent reaction (say, unfolding) is studied, the pathways of these two studied reactions can be different, of course.)

**Figure 2:**
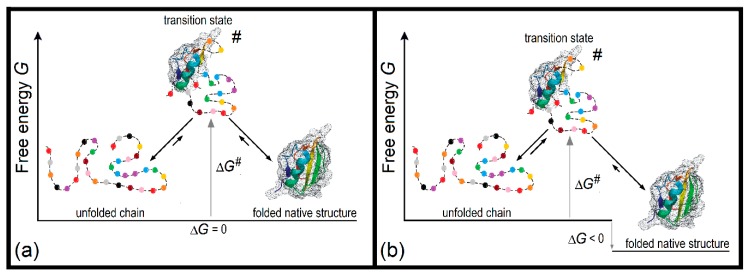
A schematic view of a sequential reversible folding–unfolding pathway of a globular protein [50,51]. At each step in the direction of folding, an unfolded residue joins the growing native structure. The native-like part (shaded) of semi-folded intermediates on the fast folding–unfolding pathway should be compact, i.e., the boundary separating unfolded (depicted with the broken line), and native phases should be minimal. The free energies of natively folded, unfolded, and transition states, which determine the folding/unfolding rates, depend on ambient conditions, but, being functions of the states, they are independent of how protein arrives at these states. ∆*G***^#^**, the transition state free energy (i.e., that of the nucleation of the folded structure) is counted off that of the initial unfolded state; it corresponds to the maximal free energy on the fastest folding–unfolding pathway. (**a**) The mid-transition case: Equal stabilities of the unfolded and native states (i.e., Δ*G* = 0) maintain dynamic (50%:50%) equilibrium between them. (**b**) The in-water case: The native state is more stable than the unfolded one (i.e., Δ*G* < 0), and therefore all or nearly all protein molecules obtain their folded native state. Adapted from [52] with some modifications.

The scheme of free energy picture looks symmetrical at the mid-transition point (Figure 2a). Indeed, there are two equally stable states with a free energy barrier between them. The height of the barrier is, therefore, the same when looking at it from both sides. Both folding and unfolding processes, under the same ambient conditions, take the same time and follow, according to the conventional “detailed balance principle” [55] the same pathway(s) and thus also look symmetrical: A protein chain tries to overcome the barrier and succeeds after millions or billions of trials. It is curious that many people have considered and discussed the Levinthal’s paradox of protein folding, but nobody has ever mentioned its symmetry to the paradox of protein **un**folding. Though, according to the conventional “detailed balance principle”, the folding and unfolding should take one and the same time at the mid-transition point! (And close times in a vicinity of this point: When the native state is more or less stable than the unfolded one by a few kcal/mol, which is typical of proteins [29], the activation barrier for folding is lower or higher than that for unfolding by a few kcal/mol as well, and the folding time is smaller or greater than the unfolding time by a few orders of magnitude [12,41], which is **nothing** as compared to those **many tens orders of magnitude** that appear in the Levinthal’s paradox.)

Using the folding–unfolding symmetry, we can consider the pathway of unfolding instead of folding to get a rough estimation of the barrier height.

In the “golf course model”, folding takes an astronomically long time because protein has to lose all its entropy before starting to gain the negative energy of interactions. For a faster folding, it is necessary that the gain of the interaction energy nearly immediately compensates the loss of conformational entropy [56].

**Figure 3:**
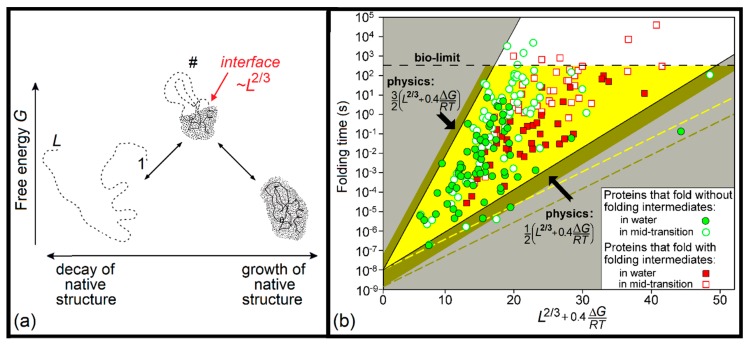
(**a**) The free energy diagram for the folding of a globular domain. Unfolded (left), native (right), and transition state (middle, #) structures are given. The dashed line denotes the unfolded phase, while the dotted body denotes the native phase. (**b**) Experimentally measured in vitro folding times at mid-transition and at “biological” (“in water”) conditions (or close to them) for 107 proteins. The bright triangular region defines the folding times at the mid-transition point outlined by physical theory (Equation (2)). If we additionally draw the biological limit line (“bio-limit”) corresponding to ~10 min, we get a “golden” triangle reflecting the physical and biological restrictions for the protein folding times at the mid-transition point. Two additional bronze areas join the “golden” triangle if we also consider physiological conditions. Two extra dashed lines define the mid-transition (yellow) and in-water (bronze) boundaries for proteins with more elongated or oblate native structures (axis ratio 2:1 or 1:2). *L* denotes the number of amino acid residues in a protein. *∆G* is the free energy difference between the native and unfolded states of the protein for particular (“in water” or “mid-transition”) conditions. Adapted from [11] with some modifications.

Such a compensation is achieved at a sequential folding–unfolding pathway, where all intermediate structures are compact (Figure 2). On the folding pathway (restored from the corresponding unfolding pathway, which is much easier to imagine), the next residue joins the growing globule losing its entropy but gaining the energy of its interactions with globule. At the end of the folding pathway, the gain of energy compensates for the loss of entropy completely; however, during the folding, this entropy-to-energy tradeoff occurs partially since the interactions of the considered residue with still unfolded part of the globule are not yet formed, thus leading to a temporary energy loss. The resulting delay is more prominent at the beginning of the folding and reaches its maximum in the transition state, being proportional to the interface between the natively folded and unfolded phases, i.e., to *L*^2/3^, where *L* is the number of amino acid residues in the protein chain. In addition to the temporary energy loss, closed loops that may protrude (see central parts of Figure 2 and Figure 3a) from the folding nucleus (corresponding to the native-like part of the transition state [12]) lead to additional temporary entropy loss, increasing the barrier height, and decelerating the folding. This looping effect is also proportional to ~*L*^2/3^, having the maximum when loops maximally cover the boundary between the native and unfolded phases in the transition state. The accurate analysis gives the following expression [11,50,51,52,57]:

*t* ~ τ × exp[(1 ± 0.5)*L*^2/3^]. (2)

The lower estimate (proportional to exp[0.5 *L*^2/3^]) concerns “simply folded” proteins where there are no closed loops protruding from the folding nucleus, and the upper estimate (proportional to exp[1.5 *L*^2/3^]) concerns proteins with complicated chain folds that inevitably have many closed loops protruding from the folding nucleus. The influence of these loops on the folding time is much greater than the influence of the chain knotting when *L* is less than ~1000 [57].

The above estimate has been obtained for the unfolded chain ↔ native globule transitions, but, because transitions between various denatured states are much less pronounced than transition between any of them and the native protein globule [40], Equation (2) should also be approximately valid for mid-transitions between the native and any denatured state of a separate protein molecule.

Thus, on the described sequential folding pathway going through compact semi-folded structures, the gain of energy nearly immediately and nearly completely compensates for the loss of entropy. The folded part is in its final (native) conformation; all chain rearrangements occur in the unfolded phase. The free energy barrier on that kind of pathway depends only on relatively subtle surface effects, being higher for the transition states having many closed loops covering boundary between the native and unfolded phases and lower in case of absence of the closed loops. The barrier scales with the number of amino acid residues *L* as *L*^2/3^.

Figure 3b shows that the deduced range of protein folding times fits well the experimental data at the above-considered mid-transition conditions (usually obtained at moderate concentrations of denaturants [12]), where the native and denatured states of proteins are equally stable. This fit remains true for proteins at more biological “in-water” conditions, where their native states are sufficiently more stable than the denatured ones (see below).

The range in the Equation (2) defines the folding times in the mid-transition point. At biological conditions, the times should be corrected depending on the stability of the native structure ∆*G* [11] (∆*G* is below zero at “biological” conditions, while ∆*G* ≡ 0 at mid-transition):

*t* ~ τ × exp[(1 ± 0.5) × (*L*^2/3^ + 0.4 × ∆*G/RT*)], (3)

where factor 0.4 reflects the fact that transition state structure has ≈40% of the native interactions, on average [11]. According to the Hammond postulate [58], the resulting stabilization of the transition state should constitute 40% of that for the native state. 

Equation (3) shows that the term ∆*G/RT* (which can reach −20 under “biological” conditions) can decrease the folding time by up to five orders of magnitude [11], in a reasonable coincidence with the experiment; these five orders of magnitude cover about a half of the “allowed” by theory folding times for proteins of *L* ~ 100 amino acid residues (see Figure 3b).

Figure 3b also shows that, in accordance with the initial assumptions done in works [50,51], there is no principal difference between folding times for the equal-size proteins that fold with or without folding intermediates [11]; earlier, it has been also shown [59] that there is no principal difference between folding times for the equal-size proteins with or without SS bonds.

Equations (2) and (3) show that all proteins of less than 80–100 residues fold sufficiently fast regardless of peculiarities of their native structures; they do not need construction of any “special pathway” to fold within a biologically-reasonable time (minutes), and thus their native 3D structures are under complete thermodynamic control. Thus, the Levinthal’s paradox is solved for the proteins of ~100 or less residues.

Figure 3b also shows that the upper limit of folding times for proteins of *L* > 100 amino acid residues corresponds, actually, not to a “physical limit” given by Equations (2) and (3), but to a “biological limit” of about 10 min under “biological” conditions.

Structures of bigger, consisting of more than ~100–200 residues, single-domain proteins, if they are too “complicated” (so that they cannot avoid formation of too many loops in their transition states), would fold significantly longer (days, weeks, etc.). These “too complicated”, as well as all “too large” (of *L* > 500 residues) quasi-spherical domains are not observed [11], and it seems that they had no chance to appear in the course of evolution. Such “structural control” of large protein domains resembles “kinetic control” proposed by Levinthal, but it works only for proteins of more than 100 residues.

It should be noted that protein chains consisting of many hundreds or thousands of amino acid residues form several domains (cf. [6,11]). Folding of multi-domain proteins in vivo usually proceeds in a domain-wise fashion [6], and although each domain can somewhat stabilize or destabilize neighboring domains, a quasi-independent (both in vivo and in vitro) folding of these domains can allow them to fold within a time typical for folding of separate domains.

## 4. Enumeration of Protein Folds

Despite the solution of the Levinthal’s paradox, some issues are still left. We can imagine unfolding as a process where fluctuating protein structure samples neighbor conformations until it occasionally samples the transition state and overcomes the free energy barrier. However, the symmetrical process of folding, though justified physically, is counterintuitive to imagine: Unfolded chain samples neighboring conformations, and after millions-to-billions fluctuations, it achieves the structure where approximately half of the protein has already folded in the native structure. It sounds a bit similar to the Levinthal-like search, so it is tempting to modify the Levinthal’s scenario to reconcile exhaustive search with reality.

As already pointed out by Levinthal, proteins obviously do not do the search at the level of individual conformations. We know from the computer experiments that each local minimum is a center of local energy funnel [36]. A protein has just to hit any conformation inside the native funnel, and after that, it immediately folds. Thus, we have to estimate the number of local minima and the time of jumping from one minimum to a neighbor.

This idea resulted in the topomer search model (TSM) proposed by Debe et al. [60] and developed further by Makarov and Plaxco [61]. TSM suggests two-stage folding: First, a protein searches randomly for the native “topomer” (cluster of states having native-like topology) and after that, quickly folds into the native structure. The estimated number of protein topomers was ~10^7^ for a 100-residue protein [60], which drastically reduced Levinthal’s zillions years to biological times.

The application of TSM to distinct protein native structures gave such good results in the estimation of protein folding times [62] that it seemed that TSM reconciles the Levinthal-like search with reality. To check this, Wallin and Chan performed a computer experiment of protein folding on lattice models with explicit chains [63] guided by the TSM implementation proposed in [61]. However, Wallin and Chan found that “finding the correct native topomer state in a random, unbiased TSM search is so unlikely that it is comparable to the hypothetical Levinthal search process.” [63].

Finkelstein and Garbuzinskiy used an alternative idea on how to cluster the protein conformational space [64,65]. They argued that each local minimum has to be more or less compact, with formed secondary structure elements. Thus, the number of local minima to enumerate is just the number of different compact folds composed by the secondary structure elements. Having considered the full combinatorics at the level of secondary structure formation, they obtained that the search time scales as

*t* ~ τ_s_⋅*L^N^* (4)

or

*t* ~ τ_s_⋅exp[*N* × ln(*L*)], (5)

where τ_s_ is some characteristic time constant associated with the secondary structure rearrangement, and *N* is the number of secondary structure elements (note that *N* is smaller than *L* by at least an order of magnitude, and ln(*L*) ≲ 5 for *L ~* 100). The value of τ_s_ (estimated from that chains of *L* ≈ 20 residues form a single α-helix within ~0.2 μs [66] and a hairpin of two β-strands within ~6 μs [67]; see also references in [11,68]) turned out to be ~10 ns, i.e., rather close to the τ value in Equations (2) and (3).

Considering a secondary structural element length as a diameter of a globule of an *L*-residue protein having a volume of ≈150 Å^3^ × *L* [46], they found that α-helix and β-strand consist of ≈3 *L*^1/3^ and ≈1.5 *L*^1/3^ residues, respectively; and the loops connecting the secondary structure elements are known to be of approximately the same size as β-strands [46], i.e., of about 1.5 *L*^1/3^ residues. Having divided the chain length *L* by the length of a secondary structure element plus a loop, they found the number of the secondary structure elements in a globule to be, at most, *N* = *L*^2/3^/3. Substituting this into Equation (5) gives the scaling for a chain of *L* ~ 100 residues (Equation (6)), which coincides with the upper estimate from the Equation (2). Note that the time in the Equations (4) and (6) gives only the upper limit of the enumeration time since many of the obtained folds do not exist in nature (such as a sole β-strand in a protein or an α-helix being in the same layer with β-strands or vice versa).

*t* ~ τ_s_⋅exp{[ln(*L*)/3] × *L*^2/3^} ≈ τ_s_⋅exp(1.5 × *L*^2/3^) (6)

Of course, this estimation does not mean that proteins fold by enumeration of the secondary structure packings. From the structure of the folding nuclei, we know that tertiary structures form concurrently with secondary structure during the unfolded chain—native structure transitions. However, it is noteworthy that the above given analysis of the Levinthal-style enumeration of secondary structure packings, resulting in Equations (4)–(6), does not contradict either to the upper estimate of the time of overcoming the free energy barrier separating the unfolded and native states of protein chain (given by Equations (2) and (3)), or to the observed time of the most slow protein folding.

## 5. Refinement of Existing Estimates of Protein Folding Times

Physical theory based on the nucleation mechanism reduced the time of searching for the global free energy minimum from Levinthal’s zillion years to milliseconds–hours. The resulting range of the folding times at mid-transition given by Equation (2) entirely agrees with the experimental data (Figure 3b), but the correlation of logarithms of the theoretically estimated and observed folding times is about 69% only. Can one develop a more accurate estimation?

This problem seems feasible because the dependence given by Equation (2) describes only the general reliance of folding time on the protein length and existing of protruding closed loops in the transition state. There is a hope that one can improve the folding time estimate by taking into account the peculiarities of the 3D native structure and/or amino acid sequence of a particular protein.

However, before that let us consider physiological conditions, which are more interesting for researchers than the mid-transition point. Up to now, we only have a stability-induced correction as an additional term in Equation (3). This correction is useful for understanding the folding time but not for its actual prediction because for most of the proteins we do not have experimentally defined stability under physiological conditions.

Thirumalai has considered downhill folding at conditions providing a high protein structure stability [69]. The folding, in this case, is decelerated only by the energy landscape ruggedness, which is proportional to *L*^1/2^. Gutin et al. carried out computer experiments at the conditions providing the fastest folding [70], i.e., also very far from the transition midpoint. They found that the folding time scales as ln *L*. Overall, this can mean that folding time can scale with *L* as *L^P^*, where *P*, depending on conditions, ranges from nearly zero [70] to 1/2 to 2/3 [50,51].

Coming back to Equation (2), recall that *L*^2/3^ reflects the surface for a general case of an *L*-residue protein. However, the shapes of real proteins can differ a lot, providing different cross-sections, even for proteins of the same size. Uniformly spherical proteins cannot avoid big boundary between the native and unfolded phases in the transition state and fold slower than elongated proteins, which can “choose” the smallest cross-section and fold faster [71] (see also [11] and dashed lines in Figure 3b).

Another way to correct the length dependencies is to recall that α-helices are very rapidly formed [66] folding units. Subtraction of the helical residues (but for those forming the first helix turn) from the protein chain length *L* gives the “effective chain length *L*_eff_” defining the protein folding time better than *L* [72].

An alternative (or rather complementary) view of helices is that they accelerate folding because they form mostly local contacts. The fraction of local contacts in a protein structure shows a good correlation with the logarithms of the folding times of small proteins having approximately one and the same size [73]. In 1998 Plaxco et al. introduced “relative contact order” (*rCO*), a general characteristic of the locality of the native contacts in protein structure: It is small for α-helical proteins (having relatively small folding time) and large for β-structural proteins (more entangled because of many long-range contacts and having relatively large folding time) [74]. The value of *rCO* discriminates well folding times for proteins of more-or-less the same size, but it fails to predict folding times of large proteins, because it depends on the chain length *L*, as approximately *L*^−1/3^ [75] (i.e., *rCO* is small for large proteins and has to predict that they fold more rapidly than small proteins, which is so far not true).

As a result, for proteins of different sizes it turned necessary to use the “absolute contact order” [75,76] (Equation (7)), which scales with *L* as about *L*^2/3^ [75], in accordance with Equation (2), and is higher for more entangled chain folds. Therefore, when *AbsCO* is used instead of (1 ± 0.5) *L*^2/3^ in Equation (2), the quality of the logarithms of folding times predictions increases from 69% to 73% [75]. What is more interesting is that when 9.5 × ln *AbsCO* (where 9.5 is an adjustable parameter) instead of *AbsCO* is used this gives even a much greater increase in correlation with logarithms of protein folding times, up to 87% for 107 proteins used in Figure 3b [77].

*AbsCO* = *L* × *rCO* (7)

The reason seems to be the presence of the biological limit, see the corresponding “boil-limit” line in Figure 3b. It cuts off the upper part of the physically allowed range, and the resulting “allowed” area for protein folding times shrinks (“golden triangle”, Figure 3b). As a result, the line corresponding to 9.5 × ln *AbsCO* + 0.4 × ∆*G/RT* can be well fitted to the beginning (up to *L* = 100) of the line ln (*t*/τ) = *L*^2/3^ + 0.4 × ∆*G/RT*, corresponding to the middle of the “golden triangle” in Figure 3b, while the rest (at *L* > 100) of the line 9.5 × ln *AbsCO* + 0.4 × ∆*G/RT* comes close to the “bio-limit” line.

At the end of this part, we should review and assess the attempts to apply machine learning techniques for a more accurate prediction of protein folding times. Machine learning has made enormous progress in different fields; see, for example, the recent improvement in protein three-dimensional structure prediction [4,5]. Machine learning relies on computer algorithms that build predictive models from experimental data without explicit instructions. Sometimes the term “machine learning methods” includes statistical methods as well; we will also use this convention henceforth. The first machine learning method for predicting protein folding times was published in 2001 [78], soon after the publication of the first empirical method for such prediction in 1998 [74].

In preparing to apply machine learning methods, an investigator must choose which protein features to use in building a given prediction method. That is, the researcher must decide which factors are potential determinants of protein folding time; this choice is entirely up to the researcher. If it becomes clear that a protein feature does not improve the prediction, or it appears to be highly similar to another feature, the researcher can remove that feature from the predictive model. For that reason, researchers often put many protein features into their initial pool of parameters and then winnow them.

Overall, machine learning methods have used features including protein length [79,80,81,82,83,84,85,86], amino acid composition [85,86,87], number of hydrophobic and charged residues [81], protein secondary structure [79,80,86,88], relative [78,80,82,89] and absolute [82] contact orders, long-range order [86,89,90], total contact distance [82,89,90], stability [78], size of amino acids, polarity, and other amino acid properties [85,89,91,92,93], among other features.

Machine learning algorithms applied in predicting protein folding times fall into three categories:

1. Multiple linear regression [79,80,81,84,85,87,88,89,91,92,93,94], in which logarithms of folding times are approximated by the sum of the protein features, with each feature having an adjusted weight. The Equation (8) below is an example of multiple linear regression.
2. Neural networks [78,83,90]. Two sources provide general descriptions of the neural networks approach [95,96].
3. Support vector machine regression [82,86]. Two sources provide general descriptions of the support vector machine approach [97,98].

Ideally, a prediction method should work for any single-domain globular protein, independently of its size, structural class, or the presence of folding intermediates [78,83,84]. However, because of importance of two-state folding proteins in studies of protein folding [12], many methods are built only for them [79,80,81,85,90,92,93].

Machine-learning methods optimize the agreement (specifically, the Pearson correlation coefficient, https://en.wikipedia.org/wiki/Pearson_correlation_coefficient) of calculated values with experimental ones. The obtained model cannot be translated to a physical model of protein folding (at least, not directly); we can only state that some protein features influence folding time more than others do. Therefore, we can discuss the performance of machine learning methods, but not their consistency with our current understanding of protein folding. The correlation coefficients between predicted and experimentally measured folding times range from 0.74 [83]—which is almost as good as that for the best empirical methods discussed above—to the amazingly high 0.99 [91].

As we can see, most machine learning methods seem to outperform analytical and empirical methods. It is, therefore, useful to recall the difference between them concerning the building of the predictive model. Analytical and empirical methods usually hypothesize a single dependence, which looks reasonable, either physically (resulting in analytical methods of prediction) or logically (resulting in empirical methods)—for example, a dependence based on contact order [74] or on chain length [51], or on contact order **and** chain length [75]. The parameters for empirical and analytical models are meaningful (e.g., contact cutoff and definition of neighbor residues in the contact order paper [74]). Empirical methods use experimental data to justify the tested hypothesis and, sometimes, to fine-tune the parameters of the model (e.g., adjustment of the contact cutoff in the contact order hypothesis [74]). However, the adjustment of the parameters is optional for the empirical methods (because the parameters have a clear physical sense); on the other hand, for machine learning methods the adjustment of the parameters plays a central role [78,79,81,82,83,87,89,90,91,92,94].

Due to the principal differences noted above, it is interesting to look at the proof of statistical significance for both empirical and machine learning methods. For a method having one hypothesis and no adjustable parameters, the correlation coefficient and *p* value are enough to elucidate its predictive power. (The *p* value is the probability of getting the obtained correlation coefficient for the given data solely by chance; *p* value < 0.05 conventionally denotes a statistically significant result.) When several hypotheses are tested, the probability of obtaining a good correlation just by chance increases correspondingly, so that the correction for multiple hypotheses should apply. In the case of adjustable parameters, to avoid overfitting, the model has to be checked against data not used for building the model.

Analytical and empirical methods usually use one hypothesis, while the adjustable parameters (if any) are few, and they are meaningful. Therefore, the correlation coefficient obtained for the whole (training) set, as well as the *p* value without correction for multiple hypotheses, are close to the correct values obtained from the testing set. In contrast, the number of generated hypotheses and the number of adjustable parameters can be enormous when researchers use machine learning methods. For example, Gromiha (who obtained the amazingly high 0.99 correlation without any attempt to correct the *p* value for using many hypotheses at once) investigated 2,138,409 hypotheses in a single study [91] and presented the three best correlations as prediction methods. In the same study, he used a pool of 49 adjustable parameters [91]; in another study, Jiang et al. used 270 features in the pool of parameters [85]. In the absence of a physically/logically reasonable idea, the formally, uncritically used “statistical proof” too often becomes the only priority for machine learning methods.

To study this phenomenon, which, in principle, can lead to drastically worse results obtained for the testing sets than those reported for the training sets, Corrales et al. checked three machine learning methods by applying them to new data, data not used when building the models [99]. It turned out that for all three considered machine learning methods the obtained correlations were significantly worse than those declared in the original publications. In all three cases, we see an overtraining in a general sense, meaning that the correlation obtained on the training set was substantially better than that for the testing set. The reason for the overtraining is that the number of experimentally measured folding times is only a little higher than a hundred (if to exclude mutant proteins) [11,100], and a part of this limited number has to be used to compose the training and the rest for the testing set, while the number of used adjustable parameters amounted to several tens. Typically, machine learning techniques optimize these tens of parameters to achieve maximal correlation on the training set, though, having a low amount of available data, this sometimes may be done only at the cost of dropping the predictive power on the testing set.

To illustrate the problem of the low amount of available data, Corrales et al. built the model having 19 adjustable parameters based on the amino acid occurrences in proteins having different folding times [99]:

ln *t* = *a_0_* + λΣ*^20^_i_*_=1_*a_i_ N_i_*, (8)

where *N_i_* is the number of amino acid residues of type *i* in the given protein sequence, and *λ*, *a_0_*, *a_1_*, …, *a_20_* are parameters fitted from the experimental data (note that *a_1_* + … + *a_20_* ≡ 1 by definition, and the values of *λ* and *a_0_* do not influence the correlation of the “predicted” (computed) and experimental ln *t* values).

**Figure 4:**
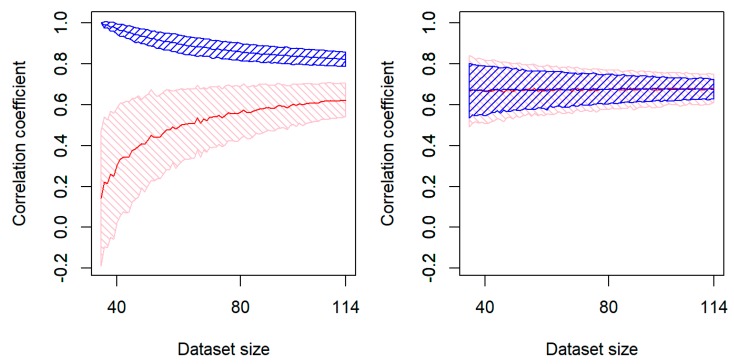
Learning curves for the model having 19 adjustable parameters based on the amino acid occurrences in proteins having different folding times (left). For sub-datasets of different sizes (*x*-axis) sampled randomly from the general experimental dataset for 114 proteins, the 60% and 40% of the sub-dataset were used for training and testing the model, respectively. The shaded area depicts the most frequently observed values of the correlation coefficient (within one standard deviation from the average value): Blue and red for the training and testing sets, respectively (left). The same for the above described (see Equation (2) and Figure 2 and Figure 3) model, where *t* = τ × exp[*L*^2/3^], that has no adjustable parameters (right). Adapted from [99] with minor modifications.

From machine learning experiments, Corrales et al. drew the so-called “learning curves” (Figure 4): From a general dataset that included 114 proteins, they first sampled a subset of a given size (from 35 to 114 proteins), see Figure 4, and then they used randomly chosen 60% of proteins from the selected subset for training the model (the aim was to adjust the variable parameters so as to obtain the highest correlation of “predictions” and experiment), while the remaining 40% of proteins were used for testing the model. They repeated this procedure thousands of times to have distributions of the correlation coefficients on the training and the testing sets.

We see that the current amount of data is far from being sufficient to get the same (or at least close) correlation coefficient on the testing and training sets (Figure 4, left panel). In contrast, the “physical” model, which follows from Equation (2) and has no adjustable parameters shows a perfect coincidence of the correlation coefficients obtained on the testing and the training sets (Figure 4, right panel).

Figure 4 shows that results obtained by machine learning techniques (with many adjustable parameters) on small training sets are very good, but then results obtained by these techniques on testing sets are very bad. When the training sets grow, results obtained on them become worse, but the really sought-for results obtained on testing sets become better. However, the results for training and testing sets can converge only when the volume of testing sets exceeds the number of adjustable parameters by at least an order of magnitude.

## 6. Conclusions

To summarize, the theoretical and semi-empirical methods (that use such meaningful parameters as the chain length [51,69,70], protein globule cross-section [71], α-helical content [72], locality of contacts [73], contact order [74,75], etc., but do not use or use a very small number of adjustable parameters) show better predictive power and correlation with experiment than the current machine learning techniques that use too many adjustable parameters (provided that correlations are obtained on testing and not training sets) [99]. Given the still relatively low number of experimental points, the purely statistical and machine learning techniques can be currently useful only for fine-tuning small second-order corrections to the existing rough but physically or biologically meaningful estimates, or for finding relatively small corrections for parameters already known to play a physically or biologically meaningful role in folding [83]. For the machine learning techniques, to predict protein folding times better than the existing theoretical and semi-empirical methods, the number of experimental points should be much higher than now.
